# PET imaging of medulloblastoma with an ^18^F-labeled tryptophan analogue in a transgenic mouse model

**DOI:** 10.1038/s41598-020-60728-6

**Published:** 2020-03-02

**Authors:** Yangchun Xin, Xuyi Yue, Hua Li, Zhiqin Li, Hancheng Cai, Arabinda K. Choudhary, Shaohui Zhang, Diane C. Chugani, Sigrid A. Langhans

**Affiliations:** 1 Katzin Diagnostic & Research PET/MR Center, Nemours/Alfred I. duPont Hospital for Children, Wilmington, DE 19803 USA; 2 Nemours Center for Childhood Cancer Research, Nemours/Alfred I. duPont Hospital for Children, Wilmington, DE 19803 USA; 3 Mayo Clinic, 4500 San Pablo Road, Jacksonville, FL 32224 USA; 4 Department of Radiology, Nemours/Alfred I. duPont Hospital for Children, Wilmington, DE 19803 USA; 5 Communication Sciences and Disorders Program, College of Health Sciences, University of Delaware, Newark, DE 19713 USA; 6 Present Address: University of Arkansas for Medical Sciences, Little Rock, AR 72205 USA; 7 Present Address: University of Delaware, Newark, DE 19713 USA; 8 Present Address: Progenics Pharmaceuticals Inc., New York, NY 10007 USA

**Keywords:** Paediatric cancer, Cancer imaging

## Abstract

*In vivo* positron emission tomography (PET) imaging is a key modality to evaluate disease status of brain tumors. In recent years, tremendous efforts have been made in developing PET imaging methods for pediatric brain tumors. Carbon-11 labelled tryptophan derivatives are feasible as PET imaging probes in brain tumor patients with activation of the kynurenine pathway, but the short half-life of carbon-11 limits its application. Using a transgenic mouse model for the sonic hedgehog (Shh) subgroup of medulloblastoma, here we evaluated the potential of the newly developed 1-(2-[^18^F]fluoroethyl)-L-tryptophan (1-L-[^18^F]FETrp) as a PET imaging probe for this common malignant pediatric brain tumor. 1-L-[^18^F]FETrp was synthesized on a PETCHEM automatic synthesizer with good chemical and radiochemical purities and enantiomeric excess values. Imaging was performed in tumor-bearing Smo/Smo medulloblastoma mice with constitutive actvation of the Smoothened (Smo) receptor using a PerkinElmer G4 PET-X-Ray scanner. Medulloblastoma showed significant and specific accumulation of 1-L-[^18^F]FETrp. 1-L-[^18^F]FETrp also showed significantly higher tumor uptake than its D-enantiomer, 1-D-[^18^F]FETrp. The uptake of 1-L-[^18^F]FETrp in the normal brain tissue was low, suggesting that 1-L-[^18^F]FETrp may prove a valuable PET imaging probe for the Shh subgroup of medulloblastoma and possibly other pediatric and adult brain tumors.

## Introduction

Brain tumors remain some of the most challenging and difficult to treat tumors in children. Despite multimodal therapy and advanced therapeutics, children diagnosed with brain tumors still have a poorer prognosis than many other pediatric cancer patients^1,2^. Among pediatric brain tumors, medulloblastoma is the most common malignant form. The tumors form in the cerebellum and tend to spread through the cerebrospinal fluid to other parts of the central nervous system (CNS) and, at times, may metastasize to other parts of the body. In recent years, great progress has been made in molecular subgrouping and risk-stratification of these tumors by genomic and proteomic analysis. Current consensus divides medulloblastoma into four major subgroups, wingless (WNT), sonic hedgehog (Shh), Group 3 and Group 4, and defines clinically relevant patient subsets^3^. WNT and Shh tumors are primarly driven by mutations leading to the activation of wingless and sonic hedgehog signaling pathways, respectively; whereas the genetics and biology underlying Group 3 and Group 4 medulloblastoma remain less well defined. While magnetic resonance imaging (MRI) may predict molecular subgroups of medulloblastoma based on tumor location and enhancement pattern to some extent^4,5^, major challenges remain in the early detection of medulloblastoma formation, metastasis, and tumor recurrence, warranting the development of non-invasive imaging modalities that can detect tumors with high sensitivity and specificity.

Multimodal brain imaging integrating MRI, computer tomography (CT) and positron emission tomography (PET), is a pivotal aid in the diagnosis of brain tumors^6,7^. Complementing each other, MRI and CT scans provide structural information, while PET allows for an important quantitative and highly sensitive metabolic assessment enabling early diagnosis of tumors and prediction of response to therapy^8^. In the clinic, [^18^F]fluorodeoxyglucose (FDG) PET has become a routine procedure for evaluating the glucose metabolism of tumors. However, the application of FDG in the diagnosis of brain tumors, and especially of low grade tumors, is limited due to the high uptake of FDG in normal grey matter. As such, a number of amino acid-based radiotracers have been developed and used for the evaluation of amino acid-involved biological process in tumors^8^. Among them, tryptophan-based PET tracers show great promise for brain tumor imaging^9–25^.

**Figure 1:**
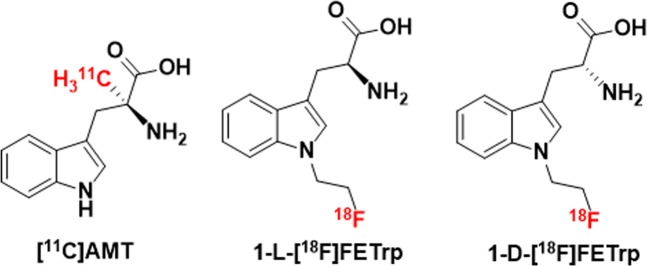
Chemical structures of [^11^C]AMT, 1-L-[^18^F]FETrp, and 1-D-[^18^F]FETrp.

Tryptophan is an amino acid that is mainly metabolized via the kynurenine pathway (KP) and within this pathway, indoleamine 2,3-dioxygenase (IDO) and tryptophan 2,3-dioxygenase (TDO) are rate-limiting enzymes^26,27^. Abnormal tryptophan metabolism via the KP is an important mechanism in brain tumors^28–30^. In medulloblastoma, the KP enzymes IDO1 and/or TDO2 messenger RNA (mRNA) expression were reported to be increased in human tumor samples across all four subgroups^31^. Previously, the PET tracer α-[^11^C]methyl-L-tryptophan ([^11^C]AMT), a tryptophan analog, has been successfully used to evaluate the increased tryptophan metabolism through KP in patients with brain tumors^18–25^. However, the short half-life of the carbon-11 isotope has limited the broad applications in clinical research and significant effort has been made in developing ^18^F-labeled tryptophan tracers^15,17,32–36^. Recent *in vitro* and *in vivo* studies suggest that a novel PET tracer, 1-(2-[^18^F]fluoroethyl)-L-tryptophan (1-L-[^18^F]FETrp), might have similar transport and metabolic properties as [^11^C]AMT (Fig. 1), and could be used to assess abnormal tryptophan metabolism via the KP in brain tumors^32–34^.

While PET imaging with 1-L-[^18^F]FETrp shows promise for detecting brain tumors^32^, previous studies have been limited to mouse xenograft models using established cell lines and primary tumor cells from adult patients, not taking into account limitations imposed by the blood brain barrier (BBB). In medulloblastoma, while changes in the BBB in the WNT form allow passage of chemotherapeutic agents into the brain and have high cure rates, the BBB is intact in the chemoresistant Shh form^37^. In the present study, we evaluated 1-L-[^18^F]FETrp for PET imaging of the most common malignant pediatric brain cancer medulloblastoma in an immunecompetent, transgenic mouse model for the Shh subgroup in which tumors occur spontaneously^38^. Using the Smo/Smo mouse model with constitutive activation of the Smoothened (Smo) receptor and a model for the Shh subgroup of medulloblastoma, we show that 1-L-[^18^F]FETrp specifically accumulated in cerebellar tumors but not in normal brain. Moreover, using L- and D-enantiomers, both with good chemical and radiochemical purities as well as enantiomeric excess values of [^18^F]FETrp, we demonstrate that only 1-L-[^18^F]FETrp had high accumulation in tumors, and thus, 1-L-[^18^F]FETrp uptake in tumor-bearing Smo/Smo mice was specific. Future studies will determine whether 1-L-[^18^F]FETrp is suitable for clinical translation as a PET imaging probe for medulloblastoma and possibly other brain tumors in pediatric and adult populations.

## Results

### Uptake of 1-L-[^18^F]FETrp in mouse brain of Smo/Smo mice with established tumors

**Figure 2:**
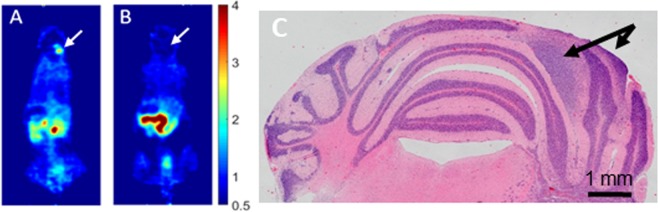
Uptake of 1-L-[^18^F]FETrp in mouse brain. (**A)** Representative coronal PET images of a Smo/Smo mouse displaying clinical signs of a tumor (white arrow). (**B**) Representative coronal PET image of an age-matched control mouse (white arrow). Intravenous injection of 1.5–2.2 MBq of 1-L-[^18^F]FETrp in 100 μL of saline was followed by image acquisition 50–60 min post-injection. (**C**) Representative image of the mouse tumor (black arrow) after Hematoxylin and Eosin (H&E) staining.

PET imaging studies on brain tumors using 1-L-[^18^F]FETrp so far have been limited to xenograft models in immunocompromised mice. Here we performed small-animal PET scans on Smo/Smo transgenic medulloblastoma mice with clinical signs of brain tumor development such as head tilt, decrease in activity, ataxia and bulging posterior skull. Age-matched Smo/Smo mice that had not yet developed tumors served as controls (3–6 months of age). Representative PET images acquired 50–60 min post injection of 1-L-[^18^F]FETrp are shown in Fig. 2A,B. The tracer highly accumulated in the cerebellar area of the tumor-bearing mouse (Fig. 2A), while in the cerebellar area of the control mouse the uptake was negligible (Fig. 2B). Mouse brains processed for histological hematoxylin and eosin (H&E) staining after PET imaging confirmed the presence of cerebellar tumors in the symptomatic mice (Fig. 2C), suggesting that 1-L-[^18^F]FETrp could serve as a PET imaging probe to detect medulloblastoma.

### 1-L-[^18^F]FETrp uptake kinetics in Smo/Smo medulloblastoma tumors

**Figure 3:**
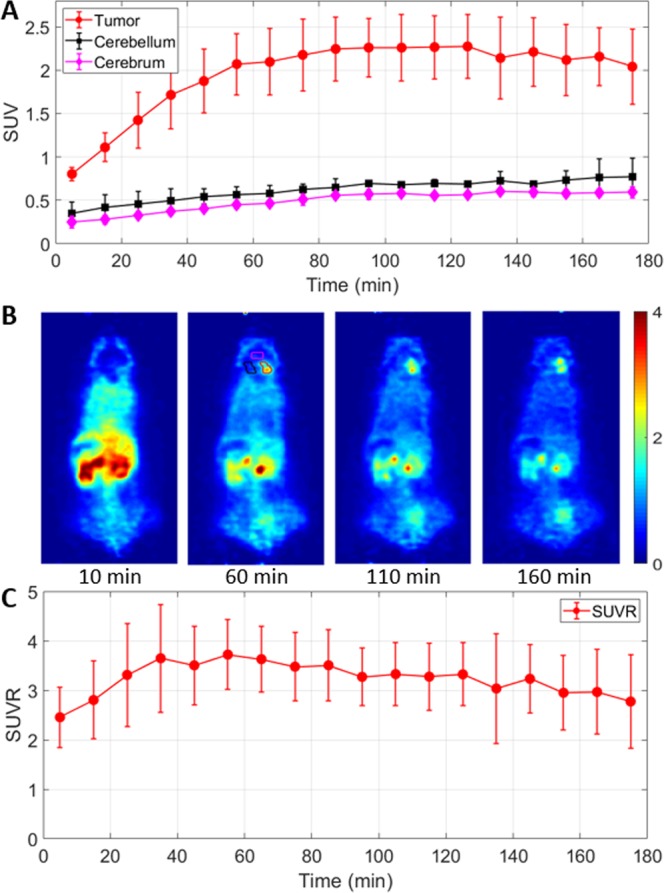
Uptake kinetics of 1-L-[^18^F]FETrp in medulloblastoma mice. **(A**)Time–activity curves of 1-L-[^18^F]FETrp uptake in brain tumor, contralateral cerebellum, and cerebrum (n = 3, p < 0.05 for all time points). (**B**) Representative axial PET images at different time points show increasing uptake of the tracer in the tumor with time after injection. (**C**) Time-contrast curve (tumor-to-contralateral cerebellum ratio) of 1-L-[^18^F]FETrp. Mice were anesthetized with 0.5–2% isoflurane/oxygen for 0–180 min dynamic microPET scans, n = 3. Intravenous injection of 1.5–2.2 MBq of 1-L-[^18^F]FETrp in 100 μL of saline.

To further investigate tracer kinetics, dynamic small animal PET studies were conducted in tumor-bearing mice (n = 3). As shown in Fig. 3A,B, the standard uptake value (SUV) of 1-L-[^18^F]FETrp in the brain tumor increased quickly with a value of approximately 1.72 ± 0.39 at 35 min post injection, reached 2.10 ± 0.39 at 65 min, peaked around 90 min post injection (2.26 ± 0.34) and plateaued until around 120 min post injection. Thereafter, the tracer washed out slowly from the brain tumor (SUV at 135 min: 2.14 ± 0.47; SUV at 155 min: 2.11 ± 0.41; SUV at 175 min: 2.04 ± 0.44). The activities in tumor-distant contralateral cerebellum and the cerebrum of the same tumor mice maintained a low level (SUV <0.77) over 180 min (n = 3, *p* < 0.05). Based on these time-activity curves, the image contrast-time (tumor-to-contralateral cerebellum ratio) profile was generated (Fig. 3C). The target-to-nontarget (tumor-to-normal cerebellum) ratio increased over time and peaked at between 40 and 90 min (standard uptake value ratio (SUVR) = 3.5–3.7) resulting in a favorable contrast within this time frame. In addition, as shown in Fig. 3B, the tracer accumulated in the kidneys and bladder over time, suggesting renal excretion of this tracer. It should be noted that the skull and bone uptake maintained a low level over time (Fig. 3B), indicating that the tracer was stable with regard to *in vivo* defluorination.

### Specificity of the 1-L-[^18^F]FETrp uptake in medulloblastoma tumors

**Figure 4:**
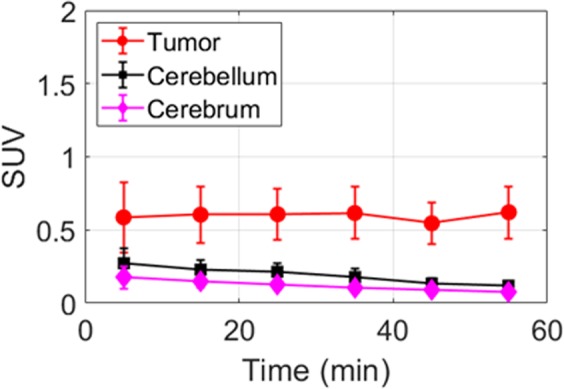
Uptake of 1-D-[^18^F]FETrp in medulloblastoma tumors. Time–activity curves of 1-D-[^18^F]FETrp uptake in different regions (tumor, cerebellum and cerebrum) in tumor bearing  mice. Mice were anesthetized with 1–2% isoflurane/oxygen for 0–60 min dynamic microPET scans, n = 3. Intravenous injection of 1.5–2.2 MBq of 1-D-[^18^F]FETrp in 100 μL of saline.

It is common that the L-enantiomer of an amino acid PET tracer has higher tumor uptake than its D-counterpart^39,40^. Consistent with this, in breast cancer, it has previously been shown that L- and D-enantiomers of 1-[^18^F]FETrp have different uptake profiles^33^. In these studies, the *in vitro* cell uptake in MDA-MB-231 cells and the *in vivo* tumor uptake in MDA-MB-231 mouse xenografts suggested that the uptake of 1-L-[^18^F]FETrp is much higher than the D-counterpart. To confirm whether the enantiomers also displayed different imaging properties in medulloblastoma, 60 min dynamic PET scans were performed with 1-D-[^18^F]FETrp in tumor-bearing Smo/Smo mice. As expected, the uptake of the D-enantiomer in nonlesioned cerebellum and cerebrum was very low in the tumor mice (Fig. 4) with an SUV <0.5 during the entire 60 min scan. In addition, the tracer uptake in tumors was also low albeit slightly higher than that in the normal brain regions (contralateral cerebellum and cerebrum). These results suggest that like breast cancer xenografts, medulloblastoma tumors preferably take up the 1-L-[^18^F]FETrp over 1-D-[^18^F]FETrp.

**Figure 5:**
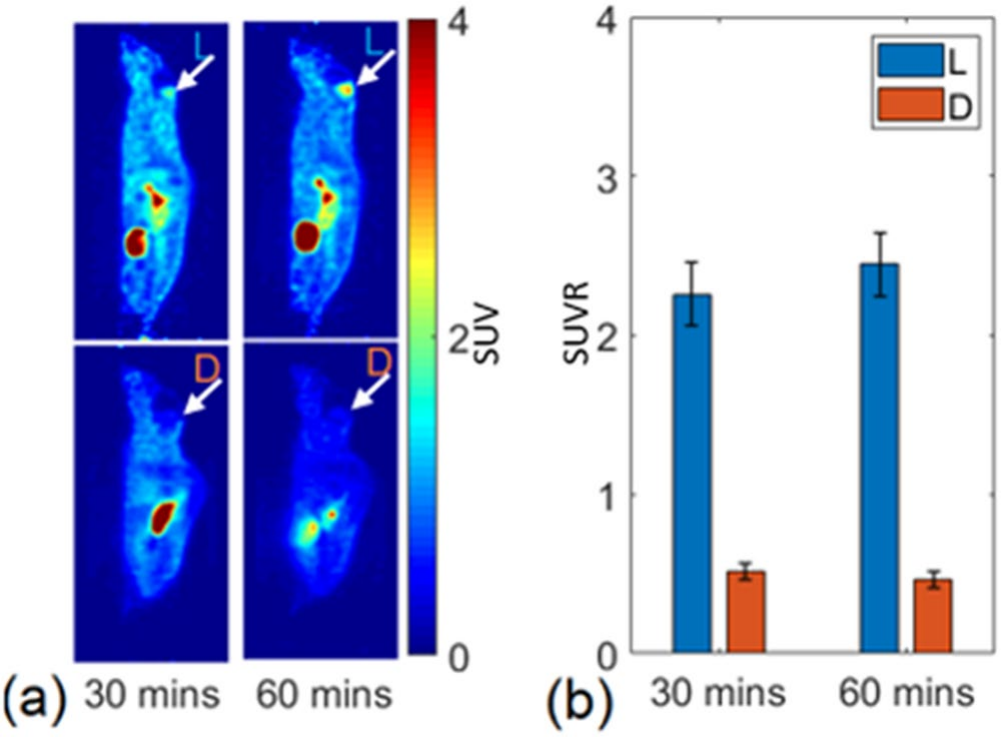
Comparison of brain uptake of 1-L- and 1-D-[^18^F]FETrp uptake in medulloblastoma in the same mouse bearing a tumor. **(a**) Top panels show sagittal images following injection of the L-enantiomer, and bottom panels show sagittal images of the same mouse following injection of the D-form. Arrows denote position of the medulloblastoma. (**b**) Standardized uptake ratios (SUVR) for regions of interest over the medulloblastoma measured at 30 and 60 min post-injection.

We further evaluated the uptake of 1-L-[^18^F]FETrp and 1-D-[^18^F]FETrp in the same tumor mouse. On day 1 we scanned a symptomatic mouse with 1-L-[^18^F]FETrp followed by a scan with 1-D-[^18^F]FETrp on the following day. As shown in Fig. 5, the tumor SUVs at 30 min (1-L-[^18^F]FETrp: 2.25; 1-D-[^18^F]FETrp: 0.52) and at 60 min (1-L-[^18^F]FETrp: 2.45; 1-D-[^18^F]FETrp: 0.41) showed significant differences between 1-L-[^18^F]FETrp and 1-D-[^18^F]FETrp over the entire period. Thus, the accumulation of 1-L-[^18^F]FETrp in medulloblastoma tumors is likely driven by intrinsic uptake mechanisms within the tumor and not an effect of general tracer leakiness into the tumor microenvironment.

## Discussion

For pediatric brain cancers, non-invasive PET imaging methods that can detect tumors with high sensitivity and specificity have lagged behind adult applications. In this study, we provide evidence that the tryptophan amino acid derivative 1-L-[^18^F]FETrp may prove suitable as a PET imaging probe for the most common malignant pediatric brain cancer medulloblastoma. Using the Smo/Smo transgenic medulloblastoma mouse model, we show that 1-L-[^18^F]FETrp can delineate cerebellar tumors with high contrast and specificity. 1-L-[^18^F]FETrp accumulation likely occurred due to active uptake by the tumor rather than simple leakiness of the BBB as the uptake of the D-enantiomer was highly reduced compared to the 1-L-[^18^F]FETrp counterpart. Further, the negligible skull uptake of 1-L-[^18^F]FETrp indicated its good *in vivo* stability towards defluorination, a desirable feature for brain tumor imaging. Defluorination would result in activity accumulation in bone, spreading radioactivity from the skull to nearby brain tissue through a partial volume effect and confounding imaging quantification. Thus, 1-L-[^18^F]FETrp may be particularly well-suited for imaging tumors in the brain.

Previous studies in breast and brain tumor xenografts demonstrated that 1-L-[^18^F]FETrp can detect abnormal tryptophan metabolism in these tumors^32,33^. However, by using xenografts, these studies did not evaluate the ability of 1-L-[^18^F]FETrp to cross the BBB and detect tumors directly in the brain, a prerequisite for brain tumor imaging in the clinic. With the use of the transgenic Smo/Smo mice in which medulloblastoma tumors occur spontaneously, we were able to use a relevant preclinical mouse model to demonstrate the ability of 1-L-[^18^F]FETrp to directly detect brain tumors with a high contrast. In addition, this well-established animal model enabled us to circumvent additional limitations of patient-derived xenograft (PDX) models, in which the interpretation of the imaging data may be compromised  due to  the use of severely immunocompromised mice. Furthermore, the inevitable local brain injury in orthotopic xenografts occurs  during the injection of the tumor cells into the brain, which may cause neuroinflammation. This is particularly important in the case of imaging probes such as the 1-L-[^18^F]FETrp, as abnormal tryptophan metabolism in tumors has been linked to the kynurenine inflammatory pathway^28,41,42^. Based on our studies in an immunocompetent, genetically engineered preclinical medulloblastoma model with spontaneous tumor occurrence and intact blood-brain barrier, we now suggest that 1-L-[^18^F]FETrp, in combination with other commonly used imaging modalities, may have clinical relevance for the diagnosis, metabolic characterization and evaluation of response to treatment of medulloblastoma.

With regard to tumor uptake, L-amino acid radiotracers in general show remarkable advantages over their corresponding D-isomer due to the preference of amino acid transporters and related enzymes for the L-isomer. As such, most amino acid PET radiotracers currently used in the clinic are L-isomers^39,40^. However, at times, D-amino acid PET radiotracers display a similar tumor uptake as their L-counterparts and in some cases, have superior contrast performance to L-isomers due to their fast clearance from the blood pool^43–45^. Furthermore, the tryptophan analog, 1-D-methyl-tryptophan (1-D-MT) has proven to be superior to its L-counterpart  and has been widely used in the clinic. Nevertheless, similar to previous findings in a breast cancer model^33,46^, our studies in Smo/Smo mice showed that the brain uptake of 1-D-[^18^F]FETrp was very low as compared to that of its L-counterpart, possibly due to preference differences in the amino acid transporter. However, it should be noted that although the tumor uptake of 1-D-[^18^F]FETrp in Smo/Smo mice was low, it was still slightly higher than that in the  normal brain regions (contralateral cerebellum and cerebrum). This could be due to increased vascularization of the tumor tissue or to a generally increased tryptophan metabolism in tumor cells as both D- and L-tryptophan can be catabolized by the KP rate-limiting enzyme IDO^47^. In medulloblastoma, IDO1 and/or TDO2 mRNA expression was reported to be increased in human tumor samples across all four subgroups^31^, but additional pathways known to affect tryptophan metabolism may induce 1-L-[^18^F]FETrp uptake in medulloblastoma as well. This includes mechanistic target of rapamycin(mTOR) signaling, a metabolic pathway that has been shown to crosstalk with the IDO1 pathway in medulloblastoma^48^. mTOR promotes medulloblastoma tumor progression^49,50^ and targeting mTOR may be a promising strategy to treat medulloblastoma^51^. Studies characterizing the molecular mechanisms and subgroup-specficity of 1-L-[^18^F]FETrp uptake in medulloblastoma are currently in progress.

## Conclusions

In this study, we investigated the utility of 1-L-[^18^F]FETrp in the Smo/Smo medulloblastoma mouse model and the preliminary data suggested that 1-L-[^18^F]FETrp is a promising PET tracer for imaging modulablastoma. Further studies on the investigation of uptake mechanism and application of this tracer to monitor tumor treatment are currently ongoing.

## Methods

### Synthesis and quality control of the PET tracers

No carrier added [^18^F]fluoride was purchased from PETNET Solutions (North Wales, PA). The radiolabeling precursors (*S*)-*tert*-butyl 2-((*tert*-butoxycarbonyl)amino)-3-(1-(2-(tosyloxy)ethyl)-1*H*-indol-3-yl)propanoate and (*R*)-*tert*-butyl 2-((*tert*-butoxycarbonyl)amino)-3-(1-(2-(tosyloxy)ethyl)-1*H*-indol-3-yl)propanoate were purchased from Affinity Research Chemicals Inc (Wilmington, DE). The radiotracer 1-L-[^18^F]FETrp was prepared using a one-pot two-step procedure on a PETCHEM automatic synthesizer following a previously reported procedure^32^. The radiotracer was purified by a semi-preparative high-performance liquid chromatography (HPLC) with an Astec CHIROBIOTIC™ T Chiral column (5 μ, 250 × 10 mm) and mobile phase of 10% ethanol in acetic acid and sodium acetate buffer (0.05 M, pH 4.0) at a flow rate of 4 mL/min. The purified radiotracer was filtered with a 0.22 μm-pore filter, giving a sterile final product of 1-L-[^18^F]FETrp for animal studies. 1-D-[^18^F]FETrp was synthesized using the same procedure but starting from the D-precursor and was purified by the same chiral HPLC column. The quality control of the synthesized radiotracer was performed on an Agilent Infinity 1260 HPLC system equipped with an Astec CHIROBIOTIC™ T Chiral column (5 μm, 250 × 4.6 mm), with a mobile phase of ethanol in water (7/3, v/v) at a flow rate of 1 mL/min through a UV detector (λ = 230 nm) followed by a radioactivity detector. The decay corrected radiochemical yields were in the range of 20–30% (n = 6). The molar activity was in the range of 15–170 GBq/µmol. The total synthesis time was about 75 min. Both the radiochemical purity and enantiomeric excess of 1-L-[^18^F]FETrp were more than 95%. The same procedure was used for the synthesis of 1-D-[^18^F]FETrp and the tracer was obtained with good radiochemical purity (>95%) and optical purity (>90%). The detailed radiolabeling and chromatographic information are included in the Supporting Information (Supplementary Figs. S1–4).

### Micro-PET imaging

For all animal studies, applicable institutional and/or national guidelines for the care and use of animals were followed and the study was approved by the Nemours Institutional Animal Care and Use Committee (IACUC). Smo/Smo mice developed in the laboratory of Dr. James Olson (Fred Hutchinson Cancer Research Center) were described previously^38^ and were obtained from the Jackson Laboratory (C57BL/6-Tg(Neurod2-Smo)A1.199Jols/J, ND2:SmoA1). Smo/Smo mice are transgenic mice that express a constitutively active point mutation SmoA1 of the Smo receptor, an integral part of the Shh pathway, under the control of the mouse Neurod2 promoter. This leads to the expression of the transgene specifically in cerebellar granule cells. Homozygous mice spontaneously form medulloblastoma tumors between three and six months of age, and the tumors have similarity to the human Shh subgroup of medulloblastoma^52^.

Micro-PET imaging was performed in Smo/Smo mice symptomatic for brain tumors (n = 9). Symptoms of brain tumor development included head tilt, decrease in activity, ataxia and bulging posterior skull. Asymptomatic littermates that had not developed brain tumors were used as control (n = 5). Imaging was performed with a PerkinElmer G4 PET-X-Ray scanner. Five min prior to imaging, the animals were anesthetized using 2–3% isoflurane in oxygen and then the mice were injected with 1.5–2.2 MBq of 1-L-[^18^F]FETrp or 1-D-[^18^F]FETrp in saline (total volume less than 100 *µ*L) *via* the tail vein. The mice were placed onto the temperature-controlled imaging bed under isoflurane anesthesia for the duration of the imaging. Dynamic imaging was performed for one hour or three hours immediately after injection.

### Image reconstruction and data analysis

Images were reconstructed on the scanner with default parameters. The duration of each frame was ten min. For the PET quantification, regions of interest (ROIs) within the tumor, the cerebellum, and the cerebrum were drawn manually on the frame 50–60 min post injection and copied to all other frames. The ROI depicting the tumor covered the tumor region with high uptake over multiple slices, while the ROI of the cerebellum was drawn on the contralateral side with a similar volume. The ROI of the cerebrum was drawn in the middle of the cerebrum with a volume of 3.65 × 3.65 × 2.28 mm^3^. ROIs were drawn in ITK-SNAP (version 3.2). Data analyses were performed in MATLAB. The resulting quantitative data were expressed in SUV, which were calculated as the ratio of regional averaged radioactivity in Becquerel per cubic centimeter to injected radioactivity in Becquerel per gram of body weight.

### Histology

After imaging, mice were euthanized, and brains were dissected, fixed with formalin, and embedded in paraffin using standard procedures. Entire brains were sectioned, and one of every ten sections from 5 µm serial sections was deparaffinized, rehydrated, and stained with H&E using standard protocols. All sections were analyzed for the presence of tumor and images were taken with a Nikon Eclipse 80i microscope.

### Statistical analysis

Data are presented as mean ± standard deviation (SD). A two-tailed paired t test was used for the comparison of the tracer uptake in the brain. A *P* value less than 0.05 was considered statistically significant.

## Supplementary information

Supplementary Material.

## References

[1] Pollack IF, Agnihotri S, Broniscer A (2019). Childhood brain tumors: current management, biological insights, and future directions. J. Neurosurg. Pediatr..

[2] Roddy E, Mueller S (2016). Late Effects of Treatment of Pediatric Central Nervous System Tumors. J. Child. Neurol..

[3] Northcott PA (2019). Medulloblastoma. Nat. Rev. Dis. Prim..

[4] Perreault S (2014). MRI surrogates for molecular subgroups of medulloblastoma. AJNR Am. J. Neuroradiol..

[5] Iv M (2019). MR Imaging-Based Radiomic Signatures of Distinct Molecular Subgroups of Medulloblastoma. AJNR Am. J. Neuroradiol..

[6] Villanueva-Meyer JE, Mabray MC, Cha S (2017). Current Clinical Brain Tumor Imaging. Neurosurg..

[7] Nandu H, Wen PY, Huang RY (2018). Imaging in neuro-oncology. Ther. Adv. Neurol. Disord..

[8] Demetriades AK, Almeida AC, Bhangoo RS, Barrington SF (2014). Applications of positron emission tomography in neuro-oncology: a clinical approach. Surg..

[9] Chiotellis A (2014). Synthesis and biological evaluation of (18)F-labeled Fluoroethoxy tryptophan analogues as potential PET tumor imaging agents. Mol. Pharm..

[10] Giglio BC (2017). Synthesis of 5-[(18)F]Fluoro-alpha-methyl Tryptophan: New Trp Based PET Agents. Theranostics.

[11] He S (2013). Radiosynthesis and biological evaluation of 5-(3-[^18^F]fluoropropyloxy)-L-tryptophan for tumor PET imaging. Nucl. Med. Biol..

[12] Huang X, Xiao X, Gillies RJ, Tian H (2016). Design and automated production of ^11^C-alpha-methyl-l-tryptophan (^11^C-AMT). Nucl. Med. Biol..

[13] Li R (2010). Synthesis and evaluation of l-5-(2-[(18)F]fluoroethoxy)tryptophan as a new PET tracer. Appl. Radiat. Isot..

[14] Nordeman P (2018). Automated GMP-production of alpha-[(11) C]methyl-L-tryptophan using a tracer production system (TPS). J. Label. Comp. Radiopharm..

[15] Sun T (2012). Radiosynthesis of 1-[^18^F]fluoroethyl-L-tryptophan as a novel potential amino acid PET tracer. Appl. Radiat. Isot..

[16] Tang T (2017). Preparation and evaluation of L- and D-5-[(18)F]fluorotryptophan as PET imaging probes for indoleamine and tryptophan 2,3-dioxygenases. Nucl. Med. Biol..

[17] Zlatopolskiy BD (2018). Discovery of 7-[(18)F]Fluorotryptophan as a Novel Positron Emission Tomography (PET) Probe for the Visualization of Tryptophan Metabolism *in Vivo*. J. Med. Chem..

[18] Juhasz C (2006). *In vivo* uptake and metabolism of alpha-[^11^C]methyl-L-tryptophan in human brain tumors. J. Cereb. Blood Flow. Metab..

[19] Juhasz C (2011). Differential kinetics of alpha-[^11^C]methyl-L-tryptophan on PET in low-grade brain tumors. J. Neurooncol.

[20] Juhasz C (2012). Quantitative PET imaging of tryptophan accumulation in gliomas and remote cortex: correlation with tumor proliferative activity. Clin. Nucl. Med..

[21] Batista CE (2009). Imaging correlates of differential expression of indoleamine 2,3-dioxygenase in human brain tumors. Mol. Imaging Biol..

[22] Peng F (2007). Assessment of progression and treatment response of optic pathway glioma with positron emission tomography using alpha-[(11)C]methyl-L-tryptophan. Mol. Imaging Biol..

[23] Alkonyi B (2012). Accurate differentiation of recurrent gliomas from radiation injury by kinetic analysis of alpha-^11^C-methyl-L-tryptophan PET. J. Nucl. Med..

[24] Jeong JW (2015). Multi-modal imaging of tumor cellularity and Tryptophan metabolism in human Gliomas. Cancer Imaging.

[25] Lukas RV (2019). Imaging tryptophan uptake with positron emission tomography in glioblastoma patients treated with indoximod. J. Neurooncol.

[26] Dounay AB, Tuttle JB, Verhoest PR (2015). Challenges and Opportunities in the Discovery of New Therapeutics Targeting the Kynurenine Pathway. J. Med. Chem..

[27] Vecsei L, Szalardy L, Fulop F, Toldi J (2013). Kynurenines in the CNS: recent advances and new questions. Nat. Rev. Drug. Discov..

[28] Adams S (2012). The kynurenine pathway in brain tumor pathogenesis. Cancer Res..

[29] Guastella AR (2016). Tryptophan PET Imaging of the Kynurenine Pathway in Patient-Derived Xenograft Models of Glioblastoma. Mol. Imaging.

[30] Bosnyak E (2015). Molecular imaging correlates of tryptophan metabolism via the kynurenine pathway in human meningiomas. Neuro Oncol..

[31] Panosyan EH, Lin HJ, Koster J, Lasky JL (2017). In search of druggable targets for GBM amino acid metabolism. BMC Cancer.

[32] Michelhaugh SK (2017). Assessment of Tryptophan Uptake and Kinetics Using 1-(2-^18^F-Fluoroethyl)-l-Tryptophan and alpha-^11^C-Methyl-l-Tryptophan PET Imaging in Mice Implanted with Patient-Derived Brain Tumor Xenografts. J. Nucl. Med..

[33] Xin Y, Cai H (2017). Improved Radiosynthesis and Biological Evaluations of L- and D-1-[(18)F]Fluoroethyl-Tryptophan for PET Imaging of IDO-Mediated Kynurenine Pathway of Tryptophan Metabolism. Mol. Imaging Biol..

[34] Henrottin J (2016). Fully automated radiosynthesis of N(1)-[(18)F]fluoroethyl-tryptophan and study of its biological activity as a new potential substrate for indoleamine 2,3-dioxygenase PET imaging. Nucl. Med. Biol..

[35] Kramer SD (2012). 5-(2-^18^F-fluoroethoxy)-L-tryptophan as a substrate of system L transport for tumor imaging by PET. J. Nucl. Med..

[36] Chiotellis A (2016). Synthesis, Radiolabeling, and Biological Evaluation of 5-Hydroxy-2-[(18)F]fluoroalkyl-tryptophan Analogues as Potential PET Radiotracers for Tumor Imaging. J. Med. Chem..

[37] Phoenix TN (2016). Medulloblastoma Genotype Dictates Blood Brain Barrier Phenotype. Cancer Cell.

[38] Hatton BA (2008). The Smo/Smo model: hedgehog-induced medulloblastoma with 90% incidence and leptomeningeal spread. Cancer Res..

[39] Filss CP, Cicone F, Shah NJ, Galldiks N, Langen KJ (2017). Amino acid PET and MR perfusion imaging in brain tumours. Clin. Transl. Imaging.

[40] Huang C, McConathy J (2013). Radiolabeled amino acids for oncologic imaging. J. Nucl. Med..

[41] Heng, B. *et al*. Understanding the role of the kynurenine pathway in human breast cancer immunobiology. *Oncotarget* **7** (2016). 10.18632/oncotarget.6467 PMC4872729 26646699

[42] Sforzini, L., Nettis, M. A., Mondelli, V. & Pariante, C. M. Inflammation in cancer and depression: a starring role for the kynurenine pathway. *Psychopharmacology (Berl)* (2019). 10.1007/s00213-019-05200-8 PMC6820591 30806743

[43] Burger IA (2014). First clinical results of (D)-^18^F-Fluoromethyltyrosine (BAY 86-9596) PET/CT in patients with non-small cell lung cancer and head and neck squamous cell carcinoma. J. Nucl. Med..

[44] Langen KJ (2005). Preferred stereoselective transport of the D-isomer of cis-4-[^18^F]fluoro-proline at the blood-brain barrier. J. Cereb. Blood Flow. Metab..

[45] Tsukada H (2006). Evaluation of D-isomers of O-^11^C-methyl tyrosine and O-^18^F-fluoromethyl tyrosine as tumor-imaging agents in tumor-bearing mice: comparison with L- and D-^11^C-methionine. J. Nucl. Med..

[46] Xin Y (2019). Evaluation of L-1-[(18)F]Fluoroethyl-Tryptophan for PET Imaging of Cancer. Mol. Imaging Biol..

[47] Stone TW, Darlington LG (2002). Endogenous kynurenines as targets for drug discovery and development. Nat. Rev. Drug. Discov..

[48] Folgiero V (2016). IDO1 involvement in mTOR pathway: a molecular mechanism of resistance to mTOR targeting in medulloblastoma. Oncotarget.

[49] Dever DP, Opanashuk LA (2012). The aryl hydrocarbon receptor contributes to the proliferation of human medulloblastoma cells. Mol. Pharmacol..

[50] Wu CC (2017). mTORC1-Mediated Inhibition of 4EBP1 Is Essential for Hedgehog Signaling-Driven Translation and Medulloblastoma. Dev. Cell.

[51] Aldaregia J, Odriozola A, Matheu A, Garcia I (2018). Targeting mTOR as a Therapeutic Approach in Medulloblastoma. Int. J. Mol. Sci..

[52] Neumann JE, Swartling FJ, Schuller U (2017). Medulloblastoma: experimental models and reality. Acta Neuropathol..

