# Supplementary material for: PET imaging of medulloblastoma with an 18F-labeled tryptophan analogue in a transgenic mouse model
Source: Sci Rep. 2020 Mar 2;10:3800. doi: 10.1038/s41598-020-60728-6 (PMC7051973; doi:10.1038/s41598-020-60728-6)
Supplement: Supplementary file 1 — Supplementary Material. [file 41598_2020_60728_MOESM1_ESM.pdf]

Supplementary Material

PET imaging of medulloblastoma with an ^{18}F-labeled tryptophan analogue in a transgenic mouse model

Yangchun Xin[1,*], Xuyi Yue[1,*], Hua Li[1], Zhiqin Li[2], Hancheng Cai[3], Arabinda K. Choudhary[4], Shaohui Zhang[1], Diane C. Chugani[1,5], and Sigrid A. Langhans[2,#]

[1]Katzin Diagnostic & Research PET/MR Center, Nemours/Alfred I. duPont Hospital for Children, Wilmington, DE 19803, USA; [2]Nemours Center for Childhood Cancer Research, Nemours/Alfred I. duPont Hospital for Children, Wilmington, DE 19803, USA; [3]Mayo Clinic, 4500 San Pablo Road, Jacksonville, FL 32224, USA; [4]Department of Radiology, Nemours/Alfred I. duPont Hospital for Children, Wilmington, DE 19803; [5]Communication Sciences and Disorders Program, College of Health Sciences, University of Delaware, Newark, DE 19713.

*These authors contributed equally.

Present address:
AKC – University of Arkansas for Medical Sciences, Little Rock, AR 72205
DCC – University of Delaware, Newark, DE 19713
SZ – Progenics Pharmaceuticals Inc., New York, NY 10007

#Corresponding Author:
Sigrid A. Langhans, PhD, Nemours/Alfred I. duPont Hospital for Children, DuPont Experimental Station, Bldg 400, #4414; 200 Powder Mill Road, Wilmington, DE 19803, E-mail: Sigrid.Langhans@nemours.org, Phone: 302-651-6538, Fax: 302-651-4827

This file contains Supplementary Figures S1-S4

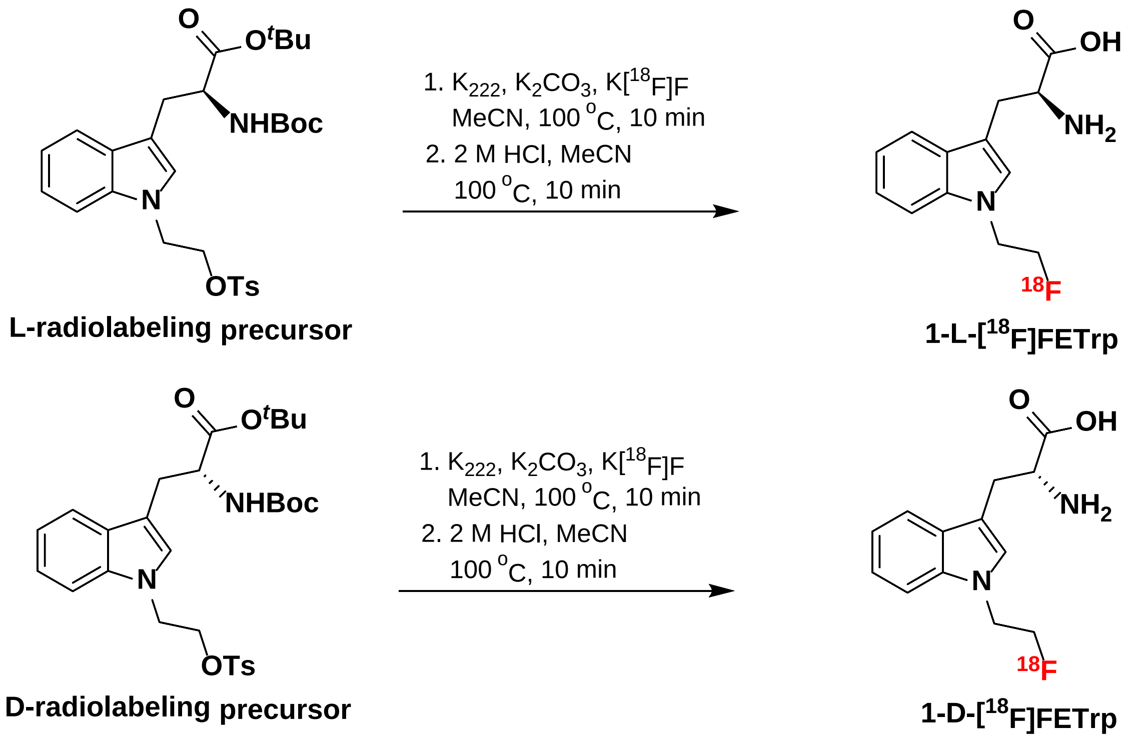

Figure S1. Radiolabeling of 1-L-[^{18}F]FETrp and 1-D-[^{18}F]FETrp.

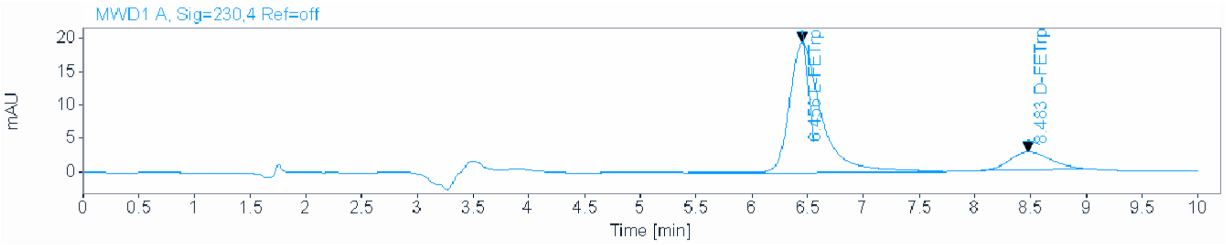

Figure S2. Representative analytical HPLC ultraviolet chromatogram of the L-FETrp and D-FETrp nonradioactive mixture. HPLC conditions: Astec CHIROBIOTIC™ T Chiral column (5 μm, 250 × 4.6 mm), EtOH in water (7/3, v/v), flow rate at 1 mL/min, UV detection wavelength at 230 nm. Retention time: L-FETrp, 6.5 min; D-FETrp, 8.5 min.

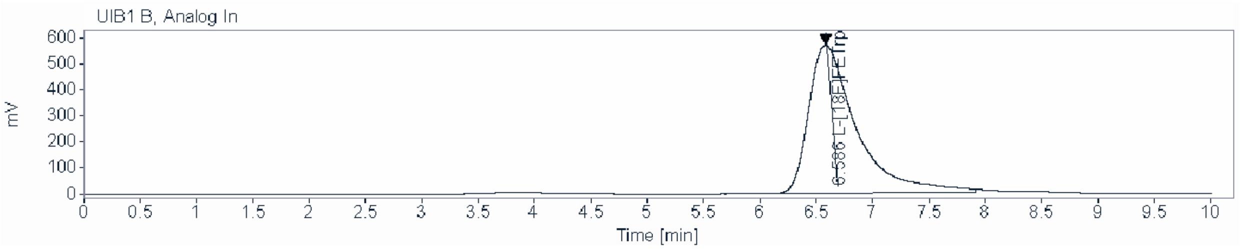

Figure S3. Representative analytical HPLC radioactive chromatogram of the purified 1-L-[^{18}F]FETrp. HPLC conditions: Astec CHIROBIOTIC™ T Chiral column (5 μm, 250 × 4.6 mm), EtOH in water (7/3, v/v), flow rate at 1 mL/min.

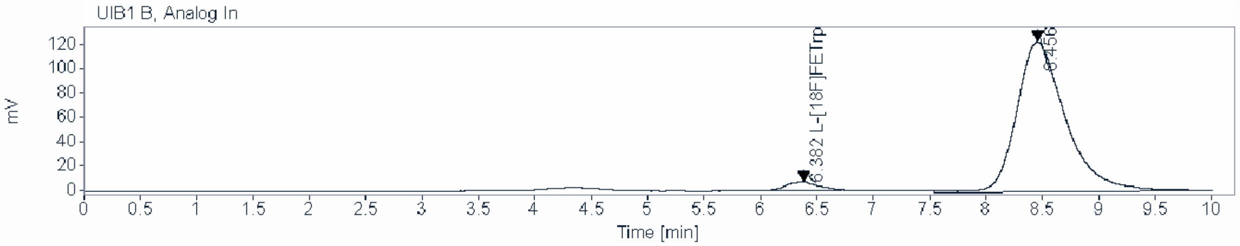

Figure S4. Representative analytical HPLC radioactive chromatogram of the purified 1-D-[^{18}F]FETrp. HPLC conditions: Astec CHIROBIOTIC™ T Chiral column (5 μm, 250 × 4.6 mm), EtOH in water (7/3, v/v), flow rate at 1 mL/min.
